# Dr. Adam Miller

**Published:** 1916-12

**Authors:** 


**Dr. Adam Miller,** Geneva 1844, died at his home in Jordanville, N. Y., Sept. 28, aged 97. He was surgeon of volunteers during the Civil War and formerly superintendent of schools for the town of Warren.

